# ER stress in Alzheimer's disease: a novel neuronal trigger for inflammation and Alzheimer's pathology

**DOI:** 10.1186/1742-2094-6-41

**Published:** 2009-12-26

**Authors:** Antero Salminen, Anu Kauppinen, Tiina Suuronen, Kai Kaarniranta, Johanna Ojala

**Affiliations:** 1 Department of Neurology, Institute of Clinical Medicine, University of Kuopio, PO Box 1627, FIN-70211 Kuopio, Finland; 2 Department of Neurology, University Hospital of Kuopio, PO Box 1777, FIN-70211 Kuopio, Finland; 3 Department of Ophthalmology, Institute of Clinical Medicine, University of Kuopio, PO Box 1627, FIN-70211 Kuopio, Finland; 4 Department of Ophthalmology, University Hospital of Kuopio, PO Box 1777, FIN-70211 Kuopio, Finland

## Abstract

The endoplasmic reticulum (ER) is involved in several crucial cellular functions, e.g. protein folding and quality control, maintenance of Ca^2+ ^balance, and cholesterol synthesis. Many genetic and environmental insults can disturb the function of ER and induce ER stress. ER contains three branches of stress sensors, i.e. IRE1, PERK and ATF6 transducers, which recognize the misfolding of proteins in ER and activate a complex signaling network to generate the unfolded protein response (UPR). Alzheimer's disease (AD) is a progressive neurodegenerative disorder involving misfolding and aggregation of proteins in conjunction with prolonged cellular stress, e.g. in redox regulation and Ca^2+ ^homeostasis. Emerging evidence indicates that the UPR is activated in neurons but not in glial cells in AD brains. Neurons display pPERK, peIF2α and pIRE1α immunostaining along with abundant diffuse staining of phosphorylated tau protein. Recent studies have demonstrated that ER stress can also induce an inflammatory response via different UPR transducers. The most potent pathways are IRE1-TRAF2, PERK-eIF2α, PERK-GSK-3, ATF6-CREBH, as well as inflammatory caspase-induced signaling pathways. We will describe the mechanisms which could link the ER stress of neurons to the activation of the inflammatory response and the evolution of pathological changes in AD.

## Introduction

Alzheimer's disease (AD) is a progressive neurodegenerative disorder involving a gradual decline in many cognitive processes ultimately leading to dementia. Accumulation of β-amyloid plaques and neurofibrillary tangles are the well-known histopathological hallmarks of AD [1,2]. It is recognized that increased production, oligomerization and aggregation of amyloid-β peptides are the crucial factors in the onset of AD. The toxic amyloid-β peptides Aβ40 and Aβ42 are processed from the APP (amyloid-β precursor protein) via cleavage by BACE1 (β-secretase) and γ-secretase complexes [1,3,4]. APP is a transmembrane protein which is folded and modified in endoplasmic reticulum (ER) and transported through the Golgi complex to the outer membrane. Once on cell surface, APP can be endocytosed and transported to the lysosomal compartment. In the brain, APP is expressed mainly in neurons but astrocytes and oligodendrocytes can also express and process APP protein. Currently, it is not clear which cellular compartment processes APP to the toxic amyloid-β peptides [3]. It seems that the processing can occur in different compartments during transport from the ER to lysosomes depending on the cellular circumstances, e.g. metabolism and stress conditions. Apparently, oligomerization of amyloid-β peptides is important since several studies have demonstrated that oligomers and fibrils are the toxic forms of amyloid-β peptides [5]. A number of studies have demonstrated that amyloid-β can be oligomerized and localized to intracellular compartments, in particular in AD [3], which can (i) disturb the function of proteasomes and lysosomes, (ii) impair calcium homeostasis and (iii) enhance the formation of neurofibrillary tangles in neurons (see below).

Accumulation of unfolded or aggregated proteins, increased oxidative stress, and metabolic disturbances are characteristic features of AD [1,3]. ER is a sensitive organelle which can recognize disturbances in cellular homeostasis and therefore it is not surprising that AD brains display many indications of ER stress [6]. ER can defend the host by activating the UPR (unfolded protein response) including signaling cascades that evoke the adaptive changes in metabolism and gene expression required to manage stress situations. Should a condition become more prolonged or overwhelming, the ER can then trigger the apoptotic program killing the cell but saving the tissue from necrotic injury. Interestingly, recent studies have demonstrated that ER stress can also elicit an innate immunity defence to protect tissues [7]. Currently, it is known that cellular stress triggers the innate immunity system by sending alarming signals, i.e. alarmins [8], or by activating the recognition receptors of inflammasomes [9]. Inflammation is a crucial event in the pathogenesis of AD, but its cause and role in AD pathogenesis are still controversial [e.g. [10]]. We will describe here the mechanisms which could link ER stress in neurons to activation of inflammatory responses and to the pathological changes observed in AD.

## ER stress and UPR signaling

**Figure 1:**
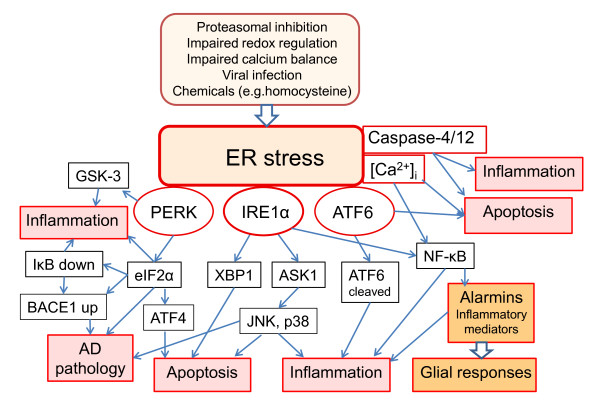
**A schematic presentation of the three branches of UPR, their signaling pathways, and pathological responses with respect to inflammation and AD pathology**.

The ER is a membrane-enclosed reticular network connecting the nuclear envelope to the Golgi complex [11]. It has multiple vital functions: (i) protein folding, post-translational modification, and transport to the Golgi complex, (ii) maintenance of cellular calcium homeostasis, (iii) synthesis of lipids and sterols, and (iv) regulation of cellular survival via a complex transducer and signaling network [11-16]. ER is a sensitive sensor of cellular homeostasis and different types of insults, e.g. proteasomal inhibition and impaired redox regulation and calcium balance, can disturb the function of ER and induce ER stress (Figure 1). ER stress involves the accumulation of unfolded and deficiently modified proteins, disturbances in lipid metabolism, and release of ER luminal Ca^2+ ^into the cytoplasm [12,13]. In particular, failure of protein quality control is detrimental to cellular survival and therefore ER can trigger an evolutionarily conserved UPR to counteract the situation [13-16].

During the last ten years, different branches of ER stress transducers have been identified and recently reviewed in detail [13-16]. Unfolded proteins are recognized in ER via three classes of sensors, (i) IRE1 (inositol-requiring protein-1), (ii) PERK (protein kinase RNA-like ER kinase), and (iii) ATF6 (activating transcription factor-6) (Figure 1). The activation mechanism of these proteins has not been totally resolved but it is known that certain molecular chaperones of ER lumen, e.g. BiP (Grp78), are involved in the activation of these transmembrane transducers. The proteins' cytoplasmic domains trigger the specific protein kinase responses or activate transcription factors which can induce the expression of hundreds of target genes. This defence response is called UPR and consists of several chaperone proteins and the proteins of antiapoptotic and antioxidative system. Excessive and prolonged ER stress can ultimately activate apoptotic cell death (Figure 1).

The activation of the IRE1 pathway triggers the dimerization of IRE1α proteins which stimulates the cytoplasmic kinase domains and subsequently the endoribonuclease domains of the IRE1α transducer [15,16] (Figure 1). The mRNA of XBP1 (X-box binding protein-1) is the only known substrate of the IRE1α endoribonuclease. IRE1α cleaves off one intron from XBP1 mRNA which activates the synthesis of XBP1 protein. XBP1 encodes the transcription factor that trans-activates expression of several UPR target genes. In addition, activated IRE1α can bind TRAF2 protein (tumor necrosis factor receptor-associated factor 2) which subsequently stimulates stress-kinases, e.g. JNK (c-Jun N-terminal kinase) [16-18], and in this way triggers apoptotic cell death [19] (Figure 1).

The lumenal domain of PERK protein is homologous to that of IRE1α, but the cytoplasmic kinase domain can phosphorylate the α-subunit of eIF2α (eukaryotic translation initiation factor-2) [15,16] (Figure 1). The phosphorylation of eIF2α protein inhibits the initiation of translation and in this way can alleviate ER stress by reducing the amount of protein transport into the ER. However, some stress-related mRNAs contain upstream sequences that can elevate translational efficiency in association with eIF2α phosphorylation during ER stress. ATF4 (activating transcription factor 4) is one of these mRNAs and the expression of ATF protein increases not only during ER stress but also in response to many other insults, e.g. hypoxia [20]. ATF4 expression can activate not only a variety of UPR genes but also genes involved in angiogenesis and autophagy [20]. Phosphorylation of eIF2α can also increase the expression of BACE1 via the unique upstream sequences in BACE1 mRNA [21].

The third branch of ER stress transducers includes the ATF6 protein and ATF-related proteins such as the bZIP transmembrane family [22] (Figure 1). Other family members are CREBH, which is expressed only in liver, OASIS, present in astrocytes, and BBF2H7, found in several tissues, e.g. neurons [22,23]. ER stress evokes the translocation of ATF6 molecules from the ER to the Golgi complex where S2P (site 2 protease) cleaves off the cytoplasmic domain which is a bZIP transcription factor. This factor is then transferred to the nucleus where it transactivates a set of UPR genes [22]. There are two ATF6 isoforms, ATF6α and ATF6β, which have opposite effects, i.e. ATF6β is a transcriptional repressor protein balancing the effect of ATF6α protein [24]. Although the functions of ATF6 branch of ER stress transducers are largely unknown, it seems that ATF6 can activate the expression of only a few UPR genes indicating that ATF6 may have functions other than those related to UPR. Doroudgar et al. [25] observed that ischemia is a sensitive inducer of the ATF6 branch of ER stress responses. Several studies have indicated that the ATF6 family of ER stress transducers can be highly specific with respect to stress signals and the expression pattern of target genes in different tissues [22].

The ER, along with mitochondria, is an important organelle in the regulation of cellular Ca^2+ ^homeostasis [12]. ER is a dynamic Ca^2+ ^store that can quickly trigger intracellular Ca^2+ ^signals but that also can serve as a Ca^2+ ^buffer by removing excessive Ca^2+ ^ions from the cytoplasm. In addition, several ER chaperones and foldases are Ca^2+^-binding proteins. This folding of proteins requires an oxidizing environment, but oxidative stress itself can induce ER stress and Ca^2+ ^release from the ER [12]. Disturbances in Ca^2+ ^regulation of ER are generally associated with ER stress, and the impaired cross talk between ER and mitochondria is a key component leading to apoptosis [16,26]. A large body of evidence has demonstrated that the Bcl-2 (B-cell lymphoma protein 2) family of antiapoptotic proteins can inhibit Ca^2+ ^release from ER in times of cellular stress, leading to apoptosis triggered by mitochondria [16,26]. Increased intracellular Ca^2+ ^concentrations can activate several signal pathways, e.g. calpains, calcineurin and phospholipases, which are related not only to apoptosis but also to a variety of physiological and pathological functions [16]. Interestingly, a number of studies have demonstrated that ER stress can activate NF-κB signaling following Ca^2+ ^release from the ER and subsequent ROS (reactive oxygen species) production, e.g. in mitochondria [27].

## ER stress in Alzheimer's disease

Neurons are vulnerable to different genetic and environmental insults which affect the homeostasis of ER function via the accumulation of unfolded proteins and disturbances in redox and Ca^2+ ^balances. Therefore, it is not surprising that a number of studies have demonstrated that ER stress is present in several neurodegenerative diseases [28-30]. Evidence of activated UPR signaling has been detected in Alzheimer's, Parkinson's and Huntington's diseases, and in ALS (amyotrophic lateral sclerosis) [6,28-32]. Furthermore, cerebral ischemia can trigger the UPR, although a concommitant drastic decline in protein synthesis clearly decreases the level of UPR [33]. Viral infections, e.g. Borna virus, can induce prominent ER stress in neurons and subsequently stimulate UPR signaling [34]. In neurons, the tubules and cisternae of ER can extend from the nuclear envelope into dendrites and dendritic spines, and also along axons as far as presynaptic terminals. This means that the neuronal ER is a very specialized organ with functionally different subcompartments [35,36]. For example, Murakami et al. [37] revealed that the ER stress response can be localized to dendrites. This heterogeneity of the ER network in neurons may be related to synaptic loss and axonal degeneration [38], particularly in the case of redox-based dysfunctions [36]. Kudo et al. [39] demonstrated that a chemical inducer of BiP chaperone expression could prevent neuronal death in both *in vitro *and *in vivo *conditions, emphasizing a role for ER stress in neuronal degeneration.

A large body of evidence indicates that the accumulation of intracellular amyloid-β and phosphorylated tau proteins, along with the perturbation of Ca^2+ ^homeostasis, plays a prominent role in the pathogenesis of AD [1,3,40]. Recently, Hoozemans et al. [41] described in detail the changes in neuronal UPR in the brains of AD patients. They observed that immunohistochemical staining of phosphorylated (activated) UPR kinases, pPERK, peIF2α and pIRE1α, was clearly increased in hippocampal neurons in AD, especially in neurons containing granulovacuolar degeneration. Interestingly, pPERK staining was abundant in neurons that showed diffuse staining for phosphorylated tau protein, whereas this staining was less prominent in neurons containing neurofibrillary tangles. The tangles themselves were not stained with pPERK. In particular, in hippocampus the CA1, CA4, and subiculum regions contained an abundance of pPERK-positive neurons. UPR markers showed a granular staining pattern that did not colocalize with ubiquitin or p62 protein, indicating that they were not aggresomes. pPERK-positive neurons also showed abundant staining for GSK-3β (glycogen synthase kinase-3β). This is an important observation since it indicates that ER stress can activate the expression of GSK-3β, a well-known tau kinase, and in that way it could enhance neurofibrillary tangle formation [42,43]. Unterberger et al. [44] have also observed that the activations of PERK, eIF2α and p38 MAPK correlate with the presence of abnormal tau in the neurons of AD patients. These immunohistochemical studies have revealed the close connection between the ER stress and tau pathology in neurons. Moreover, they demonstrated that UPR-positive staining is localized in neurons but not in glial cells.

## ER stress elicits inflammatory response

The UPR induced by ER stress involves both immediate protein kinase responses and subsequent changes in the expression of hundreds of target genes [15,16]. The purpose of these adaptive effects is to restore cellular homeostasis. However, if the provoking stress is prolonged, UPR can trigger the apoptotic cell death program inside the cell. Recent studies have demonstrated that ER stress can also generate signals which alert neighboring cells and elicit an inflammatory response to prevent more extensive tissue damage [7,45]. The ability of ER stress to induce an inflammatory response is linked to the pathogenesis of several diseases [46], e.g. obesity-induced ER stress can trigger the inflammatory response and generate peripheral insulin resistance [47]. Moreover, ER stress in liver can activate systemic inflammatory response [22] and it is suggested that ER stress can also induce autoimmune responses [48].

The inflammatory reaction is normally generated by the activation of pattern recognition receptors, e.g. TLRs (Toll-like receptors) and NLRs (NOD-like receptors) which act through signaling cascades, mostly via NF-κB system, induce an inflammatory response [49,50]. Cells suffering stress can also send alarmins which recruit and activate immune cells [8,51]. For instance, neuron-glial interactions involve several features which indicate that alarm systems are present in neurodegenerative diseases (see below). Recent studies have revealed that the transducers of ER stress can also trigger signaling pathways which are connected to the induction of inflammatory response. Next, we will discuss the latest studies demonstrating that the UPR is linked to the inflammatory response.

### IRE1-XBP1 pathway

Activation of IRE1α triggers the expression of XBP1 protein which switches on the transcription of several UPR genes but which can also stimulate phospholipid synthesis and ER biogenesis [52]. Gargalovic et al. [53] demonstrated that ER stress clearly induces the expression of IL6, IL8, MCP1 (interleukins 6 and 8, monocytes chemoattractant protein-1) and CXCL3 in endothelial cells. The depletion of XBP1 mRNA with siRNA-techniques evokes a significant inhibition in the expression level of these inflammatory mediators [53]. Furthermore, Smith et al. [54] reported that ER stress can strongly potentiate LPS (lipopolysaccharide)-induced secretion of IFN-β in macrophages. They observed that this response was dependent on the expression of XBP1 protein, although the effect is likely indirect since the promoter region of IFN-β gene does not contain any XBP1 binding sites. On the other hand, Kaser et al. [55] demonstrated that deletion of the *XBP1 *gene can induce intestinal inflammation. The genetic polymorphism of *XBP1 *observed in inflammatory bowel disease also supports these results [55]. One could argue that these observations are contradictory to the role of XBP1 in provoking inflammation but since XBP1 is a major UPR inducer its deficiency will aggravate ER stress and in that way trigger innate immunity defences. Recently, Hetz et al. [56] demonstrated that deficiency of XBP-1 protects against ALS, a devastating motor neuron disease [57] which also involves the activation of innate immunity [58]. Hetz et al. [56] used transgenic SOD1 mutant mice, a commonly studied mouse model of ALS. They observed that deletion of XBP-1 increases the level of autophagy, which reduces accumulation of aggregated SOD1 proteins and in this way protects motor neurons. However, a prolonged deficiency in UPR defence can be detrimental and induce cell injuries and inflammatory responses, as observed by Kaser et al. [55].

### IRE1-TRAF2 pathways

In 2000, Urano et al. [17] demonstrated that the cytoplasmic domain of IRE1 can recruit the TRAF-2 transducer protein, which can mediate several cellular functions, e.g. apoptosis, inflammation, and metabolism [59,60]. Later, it was observed that ASK1 (apoptosis signal-regulating kinase 1) can bind to the complex of IRE1-TRAF2, and ASK1 is an essential kinase in ER stress-induced neuronal death [61]. ASK1 is a member of the MAPKKK family which activates downstream both JNK and p38 MAP kinases and thus induces neuronal apoptosis [62] (Figure 1). Several studies have shown that the IRE1-TRAF2 pathway can activate NF-κB signaling [63-65]. Hu et al. [65] demonstrated that the IRE1-TRAF2 complex recruits the IKK (inhibitory-κB kinase) complex which binds to the TRAF2 component of the complex. The activation of IRE1 in ER stress stimulates IKK kinases which subsequently phosphorylate IκB proteins and trigger NF-κB signaling. The TRAF2 transducer can interact with several other proteins, e.g. TANK (a TRAF-binding protein) [66] and T2BP [67] which can also trigger NF-κB signaling. The NF-κB system is the major regulator of innate immunity responses in several cell types [68], but JNK can also activate inflammatory responses via the AP-1 transcription factor (activator protein-1) [69]. Interestingly, NF-κB signaling inhibits the JNK activation [70] and prevents neuronal apoptosis and tau pathology induced by the activation of JNK [62,71].

### PERK-eIF2α pathway

PERK is one of the stress kinases that can phosphorylate eIF2α protein and thus inhibit protein synthesis [72]. Hypoxia, proteasomal inhibition and ultraviolet light also inhibit protein synthesis via activation of the PERK-eIF2α axis [73-75]. All of these treatments are well-known activators of NF-κB signaling. Deng et al. [76] demonstrated that activation of NF-κB signaling correlates with reduced levels of IκBα protein during inhibition of protein synthesis. The turnover time of IκBα protein is shorter than those of NF-κB components due to a PEST sequence in IκBα protein [72,76]. This is probably a general mechanism during stress since the NF-κB system has several survival functions in host defence along with innate immunity responses, e.g. the prevention of apoptotic cell death. NF-κB signaling can repress expression of GADD153/CHOP (growth arrest and DNA damage/CEBP homology protein), which is the major pro-apoptotic transcription factor induced by ER stress [77].

### PERK-GSK-3 pathway

In addition to eIF2α, PERK has also other target proteins, e.g. GSK-3 [78] (Figure 1). GSK-3 is an interesting kinase in terms of AD pathology since it is believed to have a major role in the pathogenesis of tau-pathology [41,43]. It has been known for many years that GSK-3β can activate NF-κB signaling and enhance inflammation while lithium, a GSK-3 inhibitor, attenuates inflammatory reactions. Beurel et al. [79] have recently reviewed the crucial role of GSK-3 in the innate and adaptive immune responses. GSK-3 can regulate the function of several inflammation-associated transcription factors, e.g. NF-κB, NFAT and AP-1, but this control is clearly context-dependent [79]. Steinbrecher et al. [80] demonstrated that GSK-3 regulates in a gene-specific manner the recruitment of NF-κB complexes to cytokine promoters. For instance, IL-6 and MCP-1 require the assistance of GSK-3β for efficient transcription [80]. Demarchi et al. [81] observed that GSK-3β can inhibit the processing of NF-κB/p105 into mature p50 and thus repress NF-κB activation. It is known that GSK-3β can enhance TWEAK (TNF-like weak inducer of apoptosis)-mediated NF-κB activation in neuroblastoma cells [82]. TWEAK is believed to have a central role in NF-κB activation and neuronal death in cerebral ischemia [83].

### ATF6/CREBH pathway

The ATF6 branch of ER stress transducers is not linked to kinase signaling cascades, but trans-activates target gene expression (Figure 1). There have been very few indications that ATF6 could regulate inflammatory genes. However, Zhang et al. [84] demonstrated that in liver ER stress can activate the CREBH transcription factor which induces expression of CRP (C-reactive protein) and SAP (serum amyloid P-component). These acute-phase proteins are well-known mediators of systemic inflammatory responses. Zhang et al. [84] also claimed that activated ATF6 could interact with cleaved CREBH protein and potentiate acute-phase inflammatory responses. They also observed that proinflammatory cytokines and LPS can activate CREBH expression and in this way trigger a systemic inflammatory response. Recently, Yamazaki et al. [85] demonstrated that loss of the ER chaperone GRP78/Bip activates ATF6-dependent Akt kinase phosphorylation and subsequently triggers NF-κB signaling in NRK-52E cells. It is likely that the ATF6/CREB family of transducers has inflammatory responses but they are tissue-specific as are many other functions of ATF6 (see above).

### Inflammatory caspases

Inflammatory caspases are involved in the maturation of the IL-1 family of cytokines in protein complexes called inflammasomes [86]. This group of caspases consists of caspase 1, 4, 5, 11 (only murine), and 12. Only caspase-4 and caspase-12 are activated by ER stress but their function in ER stress is still not defined [86,87]. Several studies have indicated that activation of caspase-12 is related to ER stress-induced apoptotic cell death (Figure 1). However, the activation mechanism is still unknown although some putative mechanisms have been proposed, e.g. via Ca^2+ ^and Hip-2 signaling [88,89]. Wootz et al. [90] demonstrated that XIAP (X-linked inhibitor of apoptosis) can inhibit the activation of caspase-12 in spinal cord of ALS transgenic mice. A greater discrepancy results from the observation that there exists a polymorphism in the human caspase-12 gene that leads to the appearance of the active, ancestral full-length variant, and the inactive, truncated form of caspase-12 in the population [87,91]. It seems that carriers of truncated caspase-12 are more resistant to severe sepsis [92]. The active caspase-12 isoform attenuates inflammatory responses to endotoxin and thus can increase the risk of sepsis. Recently, LeBlanc et al. [92] demonstrated that caspase-12 inhibits activation of NF-κB signaling by binding to the NOD-Rip2 complex, which dislocates TRAF6 (TNFR-associated factor) from this complex.

Human caspase-4 and caspase-5 are orthologs of mouse inflammatory caspase-11 [86]. Caspase-4 is localized in ER and is activated by the proteolytic cleavage. Several studies have demonstrated that ER stress increases both the expression level and cleavage of caspase-4 in different experimental models [93-95]. Activation of caspase-4 can trigger either apoptosis or an inflammatory response, or both, depending on the cells involved and the experimental context (Figure 1). Further, caspase-4 is present in neurons and neuronal stress can activate this caspase and mediate apoptosis, e.g. in INCL (infantile neuronal ceroid lipofuscinosis) and probably in AD [95,96]. The activation mechanism needs to be clarified but it is known to require dimerization and interdomain processing [97] and in that respect differs from the activation of caspase-1 in inflammasomes [50]. Lakshmanan and Porter [98] observed that LPS-induced NF-κB activation is inhibited in caspase-deficient human THP1 monocytes. They demonstrated that LPS treatment induces binding of caspase-4 with TRAF6 protein and this interaction mediates NF-κB activation and subsequent cytokine secretion. TRAF-6 is an important signal transducer between several innate immunity receptors, e.g. Toll/IL-1 family, initiating NF-κB activation via the IKK complex [99]. TRAF6 protein is present in neurons and is known to mediate neurotrophin receptor p75 signaling [100] and certain inflammatory signals during hypoxia and reoxygenation in human NT2 neurons [101].

### Ca^2+^-triggered pathways

ER stress disturbs cellular Ca^2+ ^homeostasis and triggers oxidative stress [12,102]. This type of cellular stress activates the host defence response which is obviously a cell-specific reaction that includes inflammatory responses and ultimately apoptotic cell death (Figure 1). Several studies have indicated that Ca^2+ ^is an important inducer of NF-κB signaling both in ER stress [27] and in the physiological activation of neurons [103]. IP3- (inositol triphosphate-) gated Ca^2+ ^channels have a key role in calcium regulation of neurons and are probably involved in AD pathogenesis [104]. Schapansky et al. [105] demonstrated that IP3-mediated Ca^2+ ^release, stimulated by amyloid-β exposure, increases the DNA-binding activity of the NF-κB complex. Their results also indicated that activation of NF-κB signaling can inhibit the expression of CHOP (C/EBP homologous protein), a hallmark of ER-stress induced apoptosis. They also observed that the NF-κB response was even enhanced in presenilin 1-mutant neurons [105]. Nozaki et al. [77] revealed that NF-κB signaling can repress expression of CHOP after different insults which give rise to ER stress in breast cancer cells. It seems that the NF-κB activation could represent a survival effect which is linked to the UPR response. In neurons, NF-κB signaling can mediate antiapoptotic responses, increasing the expression of manganese SOD (superoxide dismutase), IAPs (inhibitors of apoptosis), and calbindin [106].

## ER stress: neuronal trigger for inflammation and AD pathology?

### Neuronal ER stress: cause or consequence of AD?

Immunohistochemical studies have revealed that neurons in postmortem brain samples of AD patients display prominent expression of markers of ER stress, e.g. pPERK, peIF2α and pIRE1α (see above). This is not a surprising result since AD involves several characteristics that could be inducers of ER stress, e.g. oxidative stress, accumulation of neurofibrillary tangles and even intraneuronal amyloid-β aggregates [1-3]. However, there is uncertainty about whether this neuronal ER stress triggers inflammation and AD pathology or whether it is a consequence of pathological processes in AD brain.

Genetic studies strongly indicate that amyloid-β production, oligomerization and aggregation have a crucial role in the pathogenesis of AD [1-5]. Recent studies have revealed that oligomers especially are the toxic form of amyloid-β in AD pathogenesis. One key question is whether synthesized APP is cleaved in ER and in this way could trigger amyloid-β oligomerization and subsequently an unfolding response in ER. BACE1 and γ-secretase are present in ER but it seems that normally amyloid-β is not cleaved in ER due to (i) the incompatible pH optimum, (ii) the presence of BACE1 stabilizers, e.g. RTN3 and Nogo-B [107], and (iii) the protective acetylation of BACE1 [108]. However, ER is a sensitive stress rheostat that is affected by a number of environmental and cellular insults. These can regulate the function of ER, e.g. by modulating expression of different chaperone molecules or by regulating the capacity for cholesterol synthesis and protein modifications. For instance, Hoshino et al. [109] demonstrated that increased ER chaperone levels can inhibit the production of amyloid-β. In addition, ER stress has been shown to increase the expression of BACE1 and thus trigger APP processing in ER (see above). Annaert et al. [110] have demonstrated that PS1 can control γ-secretase activity in pre-Golgi compartments, and PS1 mutations increase amyloid-β production in hippocampal neurons of PS1 mutant mice. Ghribi [111] has speculated on several factors that could affect amyloid-β processing in ER, such as oxidative stress, disturbances in calcium and cholesterol homeostasis, and trace metals.

ER stress and its effect on cholesterol synthesis can affect trafficking of protein molecules through the ER-Golgi pathway. It is known that seladin-1/DHCR24, a cholesterol-synthesizing enzyme in ER, modulates APP processing and amyloid-β production in vivo [112]. Greeve et al. [113] demonstrated that seladin-1 can protect against neurotoxicity induced by amyloid-β and oxidative stress. Interestingly, seladin-1 expression is selectively down-regulated in the brain regions affected in AD [113,114]. Furthermore, this reduced expression of seladin-1 is associated with increased hyperphosphorylation of tau protein and neurofibrillary tangles in AD brains [114]. A genetic study also indicated that a polymorphism in the seladin-1 gene could be associated with AD risk [115]. Recently, Sarajärvi et al. [116] demonstrated that down-regulation of seladin-1 expression does not affect APP processing in human SH-SY5Y cells over-expressing APP under normal culture conditions. However, in apoptotic conditions induced by staurosporin exposure, reduced levels of seladin-1 clearly increase BACE1 level through protein stabilization and subsequently enhanced cleavage of APP to amyloid-β peptide. They also observed that knock-down of seladin-1 expression in apoptotic conditions triggers depletion of GGA3 protein, a Golgi-localized BACE1-sorting protein, via caspase-3 cleavage. Cerebral ischemia, a typical inducer of ER stress, also induces depletion of GGA3 and increases BACE1 levels as well as production of amyloid-β [117]. Golgi transport is indispensable for APP processing, and inhibition of Golgi transport, e.g. by treatment with brefeldin A, induces a strong UPR [118]. Interestingly, during stress conditions, APP appears to accumulate in ER or Golgi complex and in this way could trigger ER stress [119].

It seems that ER stress can activate amyloid-β production (see above) but, on the other hand, secreted amyloid-β can also trigger ER stress [120]. There are clear indications that ER stress inducers have synergistic effects on levels of neuronal UPR, and ultimately can provoke apoptotic cell death [e.g. [121]]. In this respect, it is not surprising that there is evidence that viral infections may trigger AD pathology, e.g. herpes simplex virus (HSV) [122,123]. Wozniak et al. [123] demonstrated that amyloid-β accumulates in neurons infected by HSV and, unexpectedly, they observed that HSV type 1 DNA is present in amyloid plaques in AD brains. This dramatic observation still needs verification. Recently, Ishikawa and Barber [124] described an ER adaptor molecule, STING (stimulator of interferon genes), which can recognize viral infections, e.g. that of HSV, and activate innate immunity signaling via NF-κB and IRF3 pathways. The activation of STING induces expression of type I interferons, IFN-α and IFN-β, which have both beneficial and pathological effects in CNS, e.g. the overexpression of IFN-α in transgenic mice induces neurodegeneration with loss of cholinergic neurons, gliosis and angiopathy [125], which are also characteristics of AD brain. Another example of the synergistic accumulation of ER stress could be the boxing related disorder dementia pugilistica [126]. Neurotrauma is a powerful inducer of ER stress, and UPR in CNS injuries [127] could trigger AD via repeated ER stress. Ischemia and hypoxia also induce ER stress and subsequently neuronal cell death [128]. Such pathogenic mechanisms in AD, e.g. those caused by cerebral amyloid angiopathy and microhaemorrhages, could be aggravated by sustained ER stress.

In conclusion, it seems that ER stress can disturb APP processing in neurons, act synergistically with other inducers to stimulate UPR in neurons, and subsequently provoke AD pathology in the context of prolonged stress. On the other hand, AD is known to involve several pathological changes that can trigger ER stress and in that way aggravate AD pathogenesis. However, neither inflammatory disorders of the CNS nor frontotemporal tauopathies induce AD pathology, which suggests that amyloid-β has a specific role in AD pathogenesis.

### Neuronal ER stress: inducer of inflammation and ADpathology?

It is obvious that ER stress in neurons can be a crucial factor in the pathogenesis of AD. Activation of the innate immunity system and the appearance of inflammatory responses are closely involved in AD pathology [129]. As described above, there are several pathways associated with UPR that can trigger inflammatory responses as well as apoptotic cell death. There is emerging evidence that the aforementioned inducers of inflammation can be triggered inside of neurons provoked by UPR, as occurs in several other cell types. Generally, it is believed that inflammation is a secondary response to secreted amyloid-β oligomers and neuronal cell death. In addition, the role of ER stress as a causative factor of sublethal neuronal damage in AD needs to be addressed.

Innate immunity is a host defence system in tissues, principally the responsibility of specialized immune cells, e.g. microglial cells in the brain. Innate immunity can recognize PAMPs (pathogen-associated molecular patterns) and DAMPs (damage-associated molecular patterns) via PRRs (pattern recognition receptors), such as TLRs and inflammasomal receptors. We have recently reviewed the activation mechanisms of innate immunity in AD brain [129,130]. Several alarmins, i.e. messenger molecules mediating a signal of imminent danger, have been identified [8]. It is obvious that neurons can send alarming signals if there is an aggravating ER stress. Neurons also contain a large set of PRRs [129-131] of which the most interesting may well be the inflammasomal receptors, e.g. NALP1, which can activate inflammatory caspases [see our review [130]]. Several alarmins, e.g. HMGB1 [132] and S100 [133] proteins, are also expressed in neurons. A number of studies have demonstrated that during stress, neurons can express and secrete both cytokines and chemokines [e.g. [134-141]]. Chemokines, e.g. CX3CL1, CCL2, and CCL21, are important alarm molecules in neurons since, after secretion, they can activate glial chemokine receptors [134,135]. Chemokines are versatile messengers mediating neuron-glial cell interactions, e.g. they can regulate the proliferation and migration of astrocytes and microglia [134]. However, it seems that neuronal chemokines have both neuroprotective and neurotoxic effects on glial cells.

Neuronal cells can also express and secrete cytokines, e.g. IL-6 [e.g. [136,137]] and TNF-α [138,139], and even complement factors [140,141]. Recently, He et al. [142] demonstrated that deletion of TNFR1, a TNF-α responding receptor, can inhibit amyloid-β generation and prevent cognitive deficits in AD mice. This deletion reduced the expression and activity of BACE1 by blocking NF-κB signaling. Such transgenic mice also exhibit a decline in microglial activation and neuronal loss. Janelsins et al. [139] observed that chronic overexpression of neuronal TNF-α enhances local inflammatory responses in transgenic AD mice. However, there are also reports that the pro-inflammatory cytokine TNF-α has neuroprotective effects in the brain [143].

The NF-κB transcription factor system is the major signaling pathway evoking the inflammatory responses [68]. NF-κB signaling can also prevent apoptotic signals by inhibiting JNK signaling and stimulating the expression of IAP (inhibitor of apoptosis) proteins [70,144]. ER stress induces several pathways which can activate NF-κB signaling, (i) IRE1-TRAF2-IKK pathway, (ii) PERK-eIF2α activation, (iii) PERK-GSK-3 activation, and (iv) Ca^2+^-triggered pathways. There are clear indications that these pathways can induce inflammatory responses via NF-κB signaling in many different cell types (see above). In neurons, the activation of the NF-κB system is associated with several functions, e.g. neuronal survival and plasticity [145] but also with the signaling of some PRRs [129]. Ridder and Schwaninger [146] have reviewed the role of NF-κB signaling in cerebral ischemia. In AD, NF-κB signaling can enhance BACE1 expression and thus increase amyloidogenesis [147]. However, prolonged activation of NF-κB signaling, either via ER stress or inflammatory cytokines, e.g. TNF-α, can repress NF-κB signaling. Several studies have demonstrated that persistent ER stress can inhibit inflammatory responses by down-regulating IRE1-TRAF2-mediated IKK activation [65,148]. There are several other autoregulatory feedback loops involved in terminating NF-κB signaling [149]. It seems that neurons can protect themselves against overwhelming NF-κB activation by expressing Sp1-related transcription factors, which can bind to a subset of κB sites and inhibit expression of distinct target genes [150]. The expression levels of Sp3 and Sp4 factors are increased in AD brains, in particular in neurons containing tau tangles [151]. Several negative feedback systems imply that NF-κB is an important regulator in neuronal degeneration, either in inflammation or apoptosis.

Inflammatory caspases located in ER, i.e. caspase-4 and caspase-12 in humans, are another major pathway that can trigger inflammatory responses and apoptosis associated with ER stress (Figure 1, see above). As described earlier, PS1, a co-regulator of γ-secretase, is present in the ER and can control the processing of APP to amyloid-β in the ER [152]. Recently, Rechards et al. [153] demonstrated that PS1 is located in COPI-coated membranes in pre-Golgi compartments. Yukioka et al. [154] observed that AD-linked PS1 mutation can trigger the cleavage of caspase-4 and subsequently activate caspase-3 and caspase-9. This is an interesting observation since caspase-3 can cleave tau protein and induce tangle pathology [155] whereas caspase-4 itself is unable to cut tau protein [155]. Moreover, several other studies have demonstrated that exposing cells to amyloid-β induces activation of both caspase-4 and caspase-12 [89,96]. In the case of caspase-12, the amyloid-β-induced activation seems to be mediated by Hip-2 protein [89]. Moreover, in AD there seems to be a negative feedback to caspase-4 activation. The amyloidogenic APP cleavage releases AICD (APP intracellular domain) and its interacting partner, FE65 transcription factor, which subsequently can translocate to the nucleus. Kajihara et al. [156] demonstrated that this AICD-FE65 complex can bind to the promoter region of caspase-4. They also observed that FE65 factor interacts with Teashirt protein, which recruits histone deacetylases and thus inhibits the transactivation of caspase-4. Teashirt is an abundant protein in neurons. This indicates that neurons can prevent prolonged, amyloid-β-induced activation of caspase-4 and consequent inflammatory and pathological responses in AD.

The IRE1α-TRAF2-ASK1 pathway activates stress kinases [16,17] which have profound functional effects on neuronal homeostasis [19,59,62]. ER stress activates ASK1, which subsequently can trigger JNK and p38 MAPK signaling. However, it is still an open question whether ER stress is the only inducer of ASK1. For instance, Kadowaki et al. [157] observed that amyloid-β induces neuronal apoptosis through ROS-mediated ASK1 activation rather than via ER stress. However, ER stress can trigger ROS production via Ca^2+ ^mitochondrial signaling. ASK1-mediated JNK activation has great potential to provoke the pathogenesis of AD [158]. This stress kinase signaling can (i) regulate APP processing and induce accumulation of intracellular amyloid-β [159,160], (ii) phosphorylate tau protein and trigger aggregation of neurofibrillary tangles [161,162], and (iii) potentiate inflammatory responses via AP-1 activation [18]. In their histopathological study of AD brains, Lagalwar et al. [71] observed that activated pJNK accumulates in granules within hippocampal pyramidal cells. These granules frequently co-localize with granulovacuolar degeneration bodies (GVD) [71] which also contain GSK-3β [163], a well-known PERK target kinase (see above). GVD are large cytoplasmic vacuoles [71] which are reminiscent of the autophagic vacuoles commonly observed in AD [164]. Interestingly, several studies have demonstrated that excessive ER stress can induce autophagic uptake of accumulated material from the overloaded ER [165]. Activated pJNK and pGSK-3β are also present in pretangle accumulations of tau protein [166,167], which are different structures from GVDs. Sahara et al. [168] demonstrated that JNK can induce caspase cleavage of tau protein but also that GSK-3β activation is required for tau aggregation.

ER stress can cause Ca^2+ ^efflux from ER and its accumulation in mitochondria. An increased intracellular concentration of Ca^2+ ^induces several pathological responses, observed also in AD [40,169]. However, it is difficult to distinguish the specific role of ER stress in Ca^2+^-induced disturbances in pathological conditions. It seems that maintenance of Ca^2+ ^balance is important since there are Ca^2+^-sensitive modulators that can switch off stress-related signaling pathways. For instance, CIB1 (calcium and integrin binding protein 1) can bind to ASK1 and inhibit its interaction with TRAF2 [170] which blocks the stress-activated MAPK signaling pathway. Another is calnuc (nucleobindin 1) which can inhibit ATF6 activation in ER stress [171]. Calnuc, a Ca^2+^-binding protein in the Golgi complex, interacts with APP and regulates its processing [172]. Overexpression of calnuc inhibits APP expression and amyloid-β production whereas its down-regulation increases cellular levels of APP. Interestingly, levels of calnuc are significantly decreased in AD brains [172] and this could potentiate amyloid-β production.

Neuronal-glial interactions are important regulators of inflammation and AD pathology. In AD, it seems that ER stress is a greater threat to neurons than glial cells since UPR is more pronounced in neurons than glial cells (see above). Moreover, ER stress induces in neurons the production of alarmin-type of molecules, e.g. chemokines and cytokines, which affect the function of glial cells. In particular, neuronal chemokines are important messenger molecules between neurons and glial cells [134,135]. Mott et al. [173] observed that neurons can secrete CD22 protein, which inhibits proinflammatory cytokine production in microglia. Other studies have also demonstrated that neurons can suppress microglial activation [174]. This is a logical way to prevent the overactivation of microglia during stress conditions but this immunotolerance may impact detrimentally on microglial cleansing capacity and permit the aggregation of senile plaques. On the other hand, ER stress and UPR may have beneficial feedback effects since ER stress can induce expression of NF-κB inhibitors, e.g. A20, which is known to generate immunotolerance to certain non-immune cells [148] as well as to suppress the deleterious effects of excessive UPR.

## Conclusions

There are clear indications that ER stress is involved in the pathogenesis of AD. Many of the pathological characteristics of AD, e.g. oxidative stress, impaired Ca^2+ ^homeostasis, intracellular deposition of tau proteins and amyloid-β peptides, may be caused by ER stress in neurons but, on the other hand, this kind of pathology can also trigger ER stress and thus aggravate AD pathology. The markers of ER stress in AD are located in neurons rather than in glial cells. This observation is in agreement with the amyloid hypothesis of AD since misfolded and aggregated proteins are a quality problem in neurons and can trigger cellular defence through UPR. The purpose of this response is to slow down protein synthesis and increase protein folding capacity by increasing the levels of ER and cellular chaperones. In addition, UPR can strengthen the redox defence and buffering capacity of Ca^2+^, and potentiate cellular cleansing by autophagy. ER stress can also prepare neurons to undergo apoptotic cell death. Interestingly, recent studies have indicated that ER stress can also trigger inflammatory responses to defend brain tissue against necrotic injuries. This response seems to be an alarm type of response involving chemokines and cytokines to activate glial cells.

Excessive and/or prolonged ER stress can be detrimental to neurons since a delayed defence decreases the viability of neurons and can shift the UPR response to switch on an apoptotic program. However, the ER is highly specialized in neurons and the level of ER stress can vary among different subcompartments, e.g. in dendrites and axonal synapses. Initial evidence indicates that ER stress can trigger synaptic loss and axonal degeneration. In conclusion, ER stress involves all the elements that can aggravate the pathogenesis of AD.

## Competing interests

The authors declare that they have no competing interests.

## Authors' contributions

AS drafted and edited the manuscript. AK, TS, KK, JO assisted in the planning and editing of the manuscript. All authors read and approved the final manuscript.
